# The chloroplasts genomic analyses of *Rosa laevigata, R. rugosa* and *R. canina*

**DOI:** 10.1186/s13020-020-0298-x

**Published:** 2020-02-13

**Authors:** Xianmei Yin, Baosheng Liao, Shuai Guo, Conglian Liang, Jin Pei, Jiang Xu, Shilin Chen

**Affiliations:** 1 College of Pharmacy, Chengdu University of Traditional Chinese Medicine, Chengdu, 611130 China; 2 Key Laboratory of Beijing for Identification and Safety Evaluation of Chinese Medicine, Institution of Chinese Materia Medica, China Academy of Chinese Medical Sciences, Beijing, 100700 China; 3 College of Pharmacy, Shandong University of Traditional Chinese Medicine, Jinan, 250355 China

**Keywords:** *Rosa* species, cp genome, Phylogeny

## Abstract

**Background:**

Many species of the genus *Rosa* have been used as ornamental plants and traditional medicines. However, industrial development of roses is hampered due to highly divergent characteristics.

**Methods:**

We analyzed the chloroplast (cp) genomes of *Rosa laevigata, R. rugosa* and *R. canina*, including the repeat sequences, inverted-repeat (IR) contractions and expansions, and mutation sites.

**Results:**

The size of the cp genome of *R. laevigata, R. rugosa* and *R. canina* was between 156 333 bp and 156 533 bp, and contained 113 genes (30 tRNA genes, 4 rRNA genes and 79 protein-coding genes). The regions with a higher degree of variation were screened out (trnH-GUU, *trnS*-*GCU*, *trnG*-*GCC*, *psbA*-*trnH*, *trnC*-*GCA,petN*, *trnT*-*GGU*, *psbD*, *petA*, *psbJ*, *ndhF*, *rpl32,psaC* and *ndhE*). Such higher-resolution loci lay the foundation of barcode-based identification of cp genomes in *Rosa* genus. A phylogenetic tree of the genus *Rosa* was reconstructed using the full sequences of the cp genome. These results were largely in accordance with the current taxonomic status of *Rosa*.

**Conclusions:**

Our data: (i) reveal that cp genomes can be used for the identification and classification of *Rosa* species; (ii) can aid studies on molecular identification, genetic transformation, expression of secondary metabolic pathways and resistant proteins; (iii) can lay a theoretical foundation for the discovery of disease-resistance genes and cultivation of *Rosa* species.

## Background

Rosaceae is a large and diverse family with 100 genera and 3000 species. *Rosa* is a typical genus of the Rosaceae family. *Rosa chinensis* Jacq., *Rosa laevigata* Michx. and *Rosa rugosa* Thunb are documented in the 2015 version of *Chinese Pharmacopoeia* [1].

Plants of the genus *Rosa* are distributed in the temperate and subtropical regions of the Northern hemisphere [2, 3]. The genus *Rosa* has garnered increasing attention as a medicinal agent recently [4–6]. Due to the potential economic and medicinal value of peonies, it is important to understand the genetic relationships within species for future application of germplasm resources.

In conventional taxonomy, the genus *Rosa* is divided into four subgenera (*Hulthemia*, *Rosa*, *Platyrhodon*, and *Hesperhodos*), and the subgenus of *Rosa* is divided further into 10 sections (Pimpinellifoliae, Gallicanae, Caninae, Carolinae, Rosa, Synstylae, Chinenses [syn. Indicae], Banksianae, Laevigatae, and Bracteatae [7, 8].

Despite numerous recent studies examining phylogenetic relationships in the genus *Rosa*, relationships remain obscure because of: (i) hybridization in nature and in the garden, and low levels of chloroplast and nuclear genome variation [9–11]; (ii) phylogenetic analyses only based on a small number of non-coding chloroplast sequences show low internal resolution [12, 13]. *Rosa laevigata, R. rugosa* and *R. canina* have been employed in traditional Chinese medicine (TCM) formulations. However, several sympatric species of *Rosa* have been used in TCM formulations, and the diversity of medicinal materials can affect the quality and safety of medicinal materials severely.

Chloroplasts are the descendants of ancient bacterial endosymbionts. They are the common organelles of green plants, and have an essential role in photosynthesis [14]. In general, inheritance of the cp genome is patrilineal in gymnosperms, but maternal in angiosperms [15]. The cp genome is conservative in structure, contains a large single-copy (LSC) region, small single-copy (SSC) region, and two inverse repeat (IR) regions. The cp genome is an ideal research model for the study of molecular identification, phylogeny, species conservation, and genome evolution [16, 17]. Over the last decade, researchers have gained more in-depth understanding of chloroplasts, including their origin, structure, evolution, genetic engineering, as well as forward and reverse genetics [18–20]. In addition, the development of sequencing technology has greatly promoted chloroplast study [21, 22], now generating massive chloroplast genome sequence data, helping to overcome the previously unresolved relationships. Moreover, it also provides genomic information such as structure, gene order, content, and mutations in which the critical information of species identification is provided [23–27].

In previous studies, chloroplast genomes provided the effective information for identifying Rosa species [28, 29]. The chloroplast genomes of two species from the genus Rosa, *R. chinensis* and *R. rugosa, which have been* collected in Chinese Pharmacopoeia 2015 were published. In the present study, the remainder of the recorded species of the genus *Rosa* in the *Chinese Pharmacopeia*, including two used in TCM (*R. laevigata, R. rugosa)* and a traditional medicine used worldwide (*R. canina*) were identified based on the chloroplast genome. The structural characteristics, phylogenetic relationships, interspecific divergence among *R. laevigata, R. rugosa* and *R. canina* were documented.

## Materials and methods

### DNA sequencing, assembly and validation of the cp genome

The fresh leaves of *R. laevigata* and *R. rugosa* plants were collected in Shennongjia (Hubei Province, China). The dried flowers of *R canina* were purchased at a medicinal market in Beijing, China. The cetyltrimethylammonium-bromide method was used to extract the whole genomic DNA of tree peonies [30]. The DNA concentration was measured using a ND-2000 spectrometer (NanoDrop Technologies, Wilmington, DE, USA). A shotgun library (250 bp) was constructed according to manufacturer (Vazyme Biotech, Nanjing, China) instructions. Sequencing was accomplished with the X™ Ten platform (Illumina, San Diego, CA, USA) using the double terminal sequencing method (pair-end 150). The amount of raw data from the sample was 5.0 G, and > 34 million paired-end reads were attained.

Raw data were filtered by Skewer-0.2.2 [31]. Chloroplast-like reads were predicted from clean-reads by BLAST [32] searches using the sequences of the reference *Rosa chinensis*. Then, the cp reads was used to assemble sequences by SOAPdenovo-2.04 [33]. Finally, sequences were extended and gaps filled with SSPACE-3.0 and GapCloser-1.12 [34, 35]. To validate the accuracy of junction splicing, random primers were designed to test the four junctions of the sequence by polymerase chain reaction.

### Gene annotation and sequence analyses

Sequence annotation was achieved by CpGAVAS [36]. DOGMA (http://dogma.ccbb.utexas.edu/) and BLAST were used to check the results of annotation [37]. All transfer tRNA genes with default settings were detected by tRNAscanSEv1. 21 [38]. The structural features of the cp genome were drawn by OGDRAWv1.2 [39]. MEGA5.2 was used to define relative use of synonymous codons [40].

### Comparison of cp genomes

The cp genomes of *Rosa* species were completed by mVISTA [41] (Shuffle-LAGAN mode) using the genome of *R. chinensis* as the reference. Tandem Repeats Finder [42] was used to detect tandem repeats, forward repeats, and palindromic repeats as tested by REPuter [43]. Detection of simple sequence repeats (SSRs) was done by Misa.pl [44] using search parameters of mononucleotides set to ≥ 10 repeat units, dinucleotides ≥ 8 repeat units, trinucleotides and tetranucleotides ≥ 4 repeat units, and pentanucleotides and hexanucleotides ≥ 3 repeat units.

### Phylogenetic analyses

Phylogenetic trees were constructed using the genomic sequences of 21 chloroplasts. The sequences were aligned using clustalw2. Construction of an unrooted phylogenetic tree was achieved using the neighbor-joining (NJ) approach with MEGA5.2 [40] with bootstrap replicates of 1000. *Hibiscus rosa*- *sinensis* was set as the outgroup.

## Results

### DNA features of the chloroplasts of *R. laevigata*, *R. rugosa* and *R. canina*

The size of the cp genomes ranged from 156 333 bp to 156 533 bp. Among them, the largest cp genome was of *R. rugosa* (156 533 bp) and the smallest cp genome was of *R. laevigata* (156 333 bp). The total guanine + cytosine (G + C) content of the three genomes was 37.3%. *R. laevigata, R. rugosa* and *R. canina* had a cp genome with a similar structure: LSC region, SSC region, and a pair of inverted repeats (IRA/IRB). For *R. laevigata, R. rugosa* and *R. canina*, the length of the LSC region of the cp genome varied from 85 452 bp to 85 657 bp, and the G + C content from 35.2% to 35.3%; the length of SSC-region distribution was from 18 742 bp to 18 785 bp, and the G + C content was from 31.3 to 31.4%. The IR region had a length distribution from 26 048 bp to 26 053 bp, and the G + C content was 42.7% (Table 1). The DNA G + C content is an important indicator of species affinity [45], and *R. laevigata, R. rugosa* and *R. canina* have highly similar cpDNA G + C content. The DNA G + C content of the IR regions was higher than that of LSC and SSC regions, which is similar to that seen with other angiosperms [46]. In general, the relatively high DNA G + C content of the IR regions is attributable to rRNA genes and tRNA genes [47, 48]. After annotation, the sequences of the whole cp genome of *R. laevigata, R. rugosa* and *R. canina* was submitted to the National Center for Biotechnology Information database (NCBI), the GenBank accession number in Table 1.

**Table 1:** Summary of complete chloroplast chloroplast genomes for *R. laevigata, R. rugosa* and *R. canina*

Species	*R. rugosa*	*R. laevigata*	*R. cania*
Large single copy			
Length (bp)	85,657	85,452	85,653
G + C (%)	35.2	35.3	35.2
Length (%)	54.7	54.7	54.7
Small single copy			
Length (bp)	18,780	18,785	18,742
G + C (%)	31.4	31.3	31.4
Length (%)	12.0	12.0	12.0
Inverse repeat			
Length (bp)	26,048	26,048	26,053
G + C (%)	42.7	42.7	42.7
Length (%)	16.7	16.7	16.7
Total			
Length (bp)	156,533	156,333	156,501
G + C (%)	37.3	37.3	37.3
Accession number	MN661138	MN661139	MN661140

**Fig. 1:**
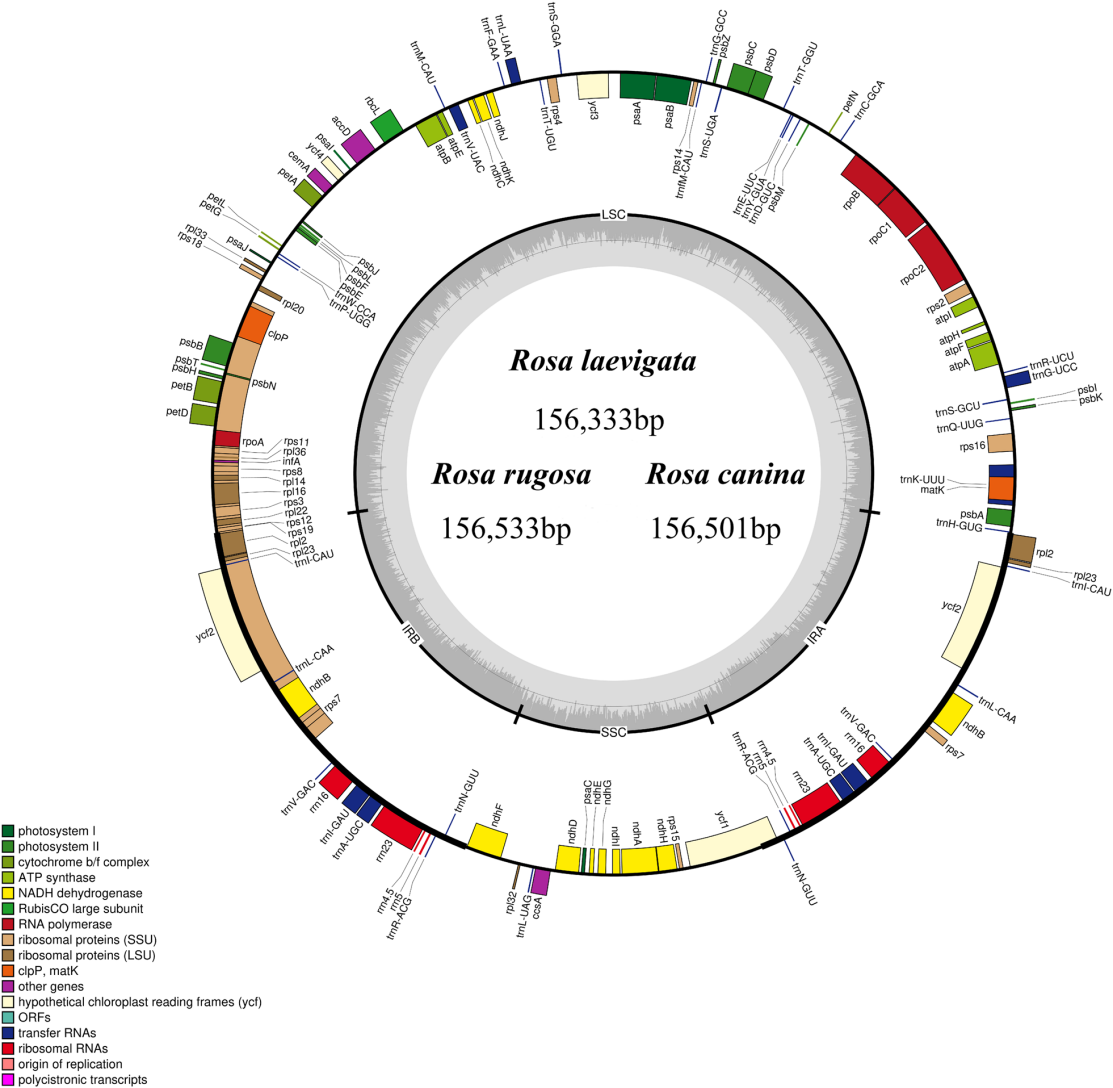
Gene map of the chloroplast genome of *R. laevigata, R. rugosa* and *R. canina*. Genes within the circle are transcribed clockwise, and those outside are transcribed counterclockwise. Genes belonging to different functional groups are color-coded. The dark-gray in the inner circle corresponds to DNA G + C content, whereas the light-gray corresponds to A + T content

A physical map of the cp genomes of *R. laevigata, R. rugosa* and *R. canina* was drawn according to annotation results using OGDraw [39] (Fig. 1). A total of 113 genes were contained in the cp genome of *R. laevigata, R. rugosa* and *R. canina*: four rRNA genes, 30 tRNA genes, and 79 protein-coding genes (Table 2). Most genes could be divided crudely into three groups: “self-replication-related”, “photosynthesis-related”, and “other” (Table 2) [49].

**Table 2:** Genes in the chloroplast genome of *R. laevigata, R. rugosa* and *R. canina*

Category	Group of genes	Name of genes
Self-replication	Large subunit of ribosomal proteins	rpl2*,a, 14, 16*, 20, 22, 23a, 33, 36
	Small subunit of ribosomal proteins	rps2, 3, 4, 7a, 8, 11, 12*,a, 14, 16*, 18, 19
	DNA-dependent RNA polymerase	rpoA, B, C1*, C2
	rRNA genes	*rrn16Sa, rrn23Sa, rrn4.5Sa, rrn5Sa*
	tRNA genes	*trnA*-UGC*,a, trnC-*GCA, trnD*-*GUC, trnE*-*UUC, trnF*-*GAA, trnfM*-*CAU, trnG*-UCC*, trnG-*GCC, trnH*-*GUG, trnI*-*CAU, trnI*-GAU*,a, trnK-UUU*, trnL-*CAA, trnL*-UAA*, trnL-*UAG, trnM*-*CAU, trnN*-*GUU, trnP*-*UGG, trnQ*-*UUG, trnR*-*ACG, trnR*-*UCU, trnS*-*GCU, trnS*-*GGA, trnS*-*UGA, trnT*-*GGU, trnT*-*UGU, trnV*-*GAC, trnV*-UAC*, trnW-*CCA, trnY*-*GUA*
Photosynthesis	Photosystem I	*psaA, B, C, I, J*
	Photosystem II	*psbA, B, C, D, E, F, H, I, J, K, L, M, N, T, Z,*
	NADH oxidoreductase	ndhA*, B*,a, C, D, E, F, G, H, I, J, K
	Cytochrome b6/f complex	petA, B*, D*, G, L, N
	ATP synthase	atpA, B, E, F*, H, I
	Rubisco	*rbcL*
Other genes	Maturase	*matK*
	Protease	clpP*
	Envelope membrane protein	*cemA*
	Subunit acetyl-CoA-carboxylase	*accD*
	c-Type cytochrome synthesis gene	*ccsA*
	Conserved open-reading frames	ycf1, 2a, 3*, 4, 15

In all anticipated genes of the cp genomes of *R. laevigata, R. rugosa* and *R. canina*, introns were discovered in 17 genes: six tRNA genes and 11 protein-encoding genes (Table 3). The tRNA genes with introns were *trnK*-*UUU, trnL*-*UAA, trnV*-*UAC, trnI*-*GAU, trnG*-*UCC* and *trnA*-*UGC*. The 11 coding genes with introns were *rps12, rps16, rpl16, rpl2, rpoC1, ndhA, ndhB, ycf3, petB, clpP* and *petD*. Three of the 17 intron-containing genes were inserted by three introns (*rps12, ycf3, clpP*). The remainder of the genes were inserted by only one intron. Of these, *trnH*-*UUU* contained the largest intron (2500 bp), which contained the whole *matK*. Similar to other angiosperms, *rps12* of chloroplasts in *R. laevigata, R. rugosa* and *R. canina* resulted from trans-splicing activity. The 5′ end of *rps12* was in the LSC region, and the 3′ end was in the IR region.

**Table 3:** Length of exons and introns in genes with introns in the chloroplast genome of three medicinal roses

Species	Gene	Location	Exon I (bp)	Intron I (bp)	Exon II (bp)	Intron II (bp)	Exon III (bp)
*R. rugosa*	*trnK*-*UUU*	LSC	35	350	37		
	*trnG*-*UCC*	LSC	23	698	48		
	*trnL*-*UAA*	LSC	35	549	50		
	*trnV*-*UAC*	LSC	35	600	39		
	*trnI*-*GAU*	IR	37	956	35		
	*trnA*-*UGC*	IR	38	814	35		
	rps12*	LSC	26	538	232		114
	*rps16*	LSC	230	881	40		
	*rpl16*	LSC	399	980	9		
	*rpl2*	IR	434	683	391		
	*rpoC1*	LSC	1611	769	453		
	*ndhA*	SSC	540	1216	552		
	*ndhB*	IR	756	678	777		
	*ycf3*	SSC	153	768	228	742	126
	*petB*	LSC	6	791	642		
	*clpP*	LSC	228	647	292	844	71
	*petD*	LSC	8	722	475		
*R. laevigata*	*trnK*-*UUU*	LSC	35	2500	37		
	*trnG*-*UCC*	LSC	23	697	48		
	*trnL*-*UAA*	LSC	35	549	50		
	*trnV*-*UAC*	LSC	35	600	39		
	*trnI*-*GAU*	IR	37	956	35		
	*trnA*-*UGC*	IR	38	816	35		
	rps12*	LSC	26	538	232		114
	*rps16*	LSC	230	872	40		
	*rpl16*	LSC	399	964	9		
	*rpl2*	IR	434	683	391		
	*rpoC1*	LSC	1611	769	453		
	*ndhA*	SSC	540	1245	522		
	*ndhB*	IR	756	678	777		
	*ycf3*	SSC	153	782	228	742	126
	*petB*	LSC	6	793	642		
	*clpP*	LSC	228	648	292	831	71
	*petD*	LSC	8	722	475		
*R. cania*	*trnK*-*UUU*	LSC	35	2500	37		
	*trnG*-*UCC*	LSC	23	697	48		
	*trnL*-*UAA*	LSC	35	548	50		
	*trnV*-*UAC*	LSC	35	600	39		
	*trnI*-*GAU*	IR	37	956	35		
	*trnA*-*UGC*	IR	38	814	35		
	rps12*	LSC	26	538	232		114
	*rps16*	LSC	230	883	40		
	*rpl16*	LSC	399	964	9		
	*rpl2*	IR	434	683	391		
	*rpoC1*	LSC	1611	700	453		
	*ndhA*	SSC	540	1205	552		
	*ndhB*	IR	756	678	777		
	*ycf3*	SSC	153	785	228	740	126
	*petB*	LSC	6	792	642		
	*clpP*	LSC	228	650	292	839	71
	*petD*	LSC	8	728	475		

### Analyses of long repetitive sequences and SSRs

**Fig. 2:**
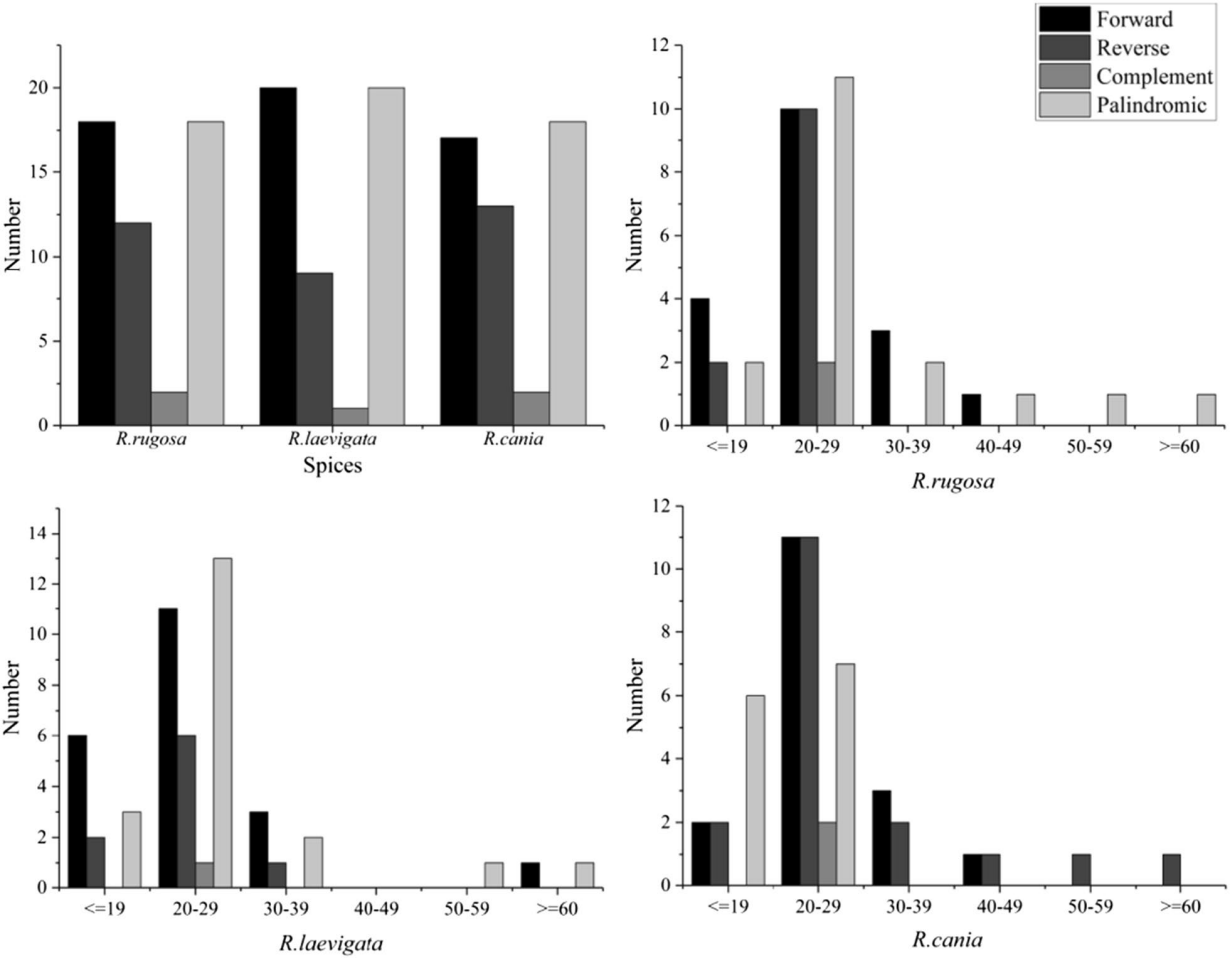
Long repetitive sequences in the chloroplast genomes of *R. laevigata, R. rugosa* and *R. canina*

For *R. laevigata, R. rugosa* and *R. canina*, interspersed repeated sequences (IRSs) were evaluated in the cp genomes with a repeat-unit length of ≥ 30 bp. These comprised forward repeats, reverse repeats, complementary repeats, and palindromic repeats. Fifty 50 IRSs were found in *R. rugosa*; 60 IRS in *R. laevigata* and 50 IRS in *R. canina*. Among all types of IRS, the sequence lengths of 20–29 bp occurred most frequently. IRS analyses of the cp genomes of *R. laevigata, R. rugosa* and *R. canina* are shown as Fig. 2.

**Fig. 3:**
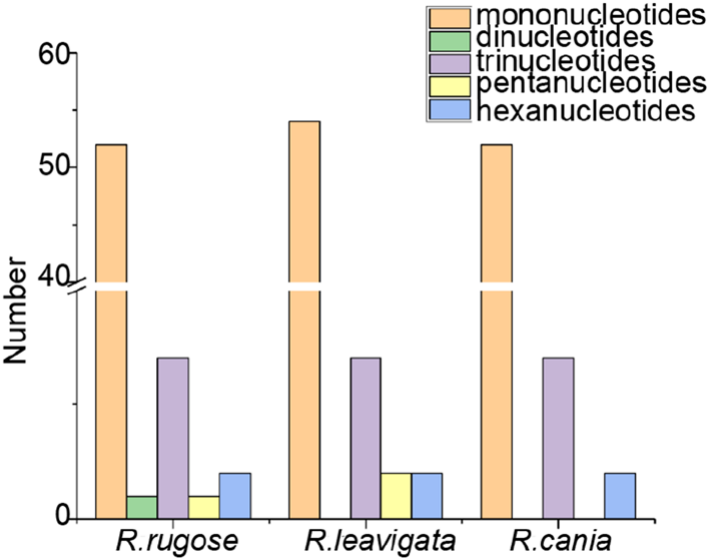
SSR distribution in the chloroplast genomes of *R. laevigata, R. rugosa* and *R. canina*

SSRs are disposed to slipped-strand mispairing, which is a key mutational mechanism for generating SSR polymorphisms [50]. SSRs at the intra-specific level in the cp genome are variable, so they are used regularly as genetic markers in studies of evolution and population genetics [51–53]. We found 63 SSRs in *R. rugosa,* 62 SSRs in *R canina,* and 65 SSRs in *R. laevigata* (Fig. 3).

### Genomic sequences

**Fig. 4:**
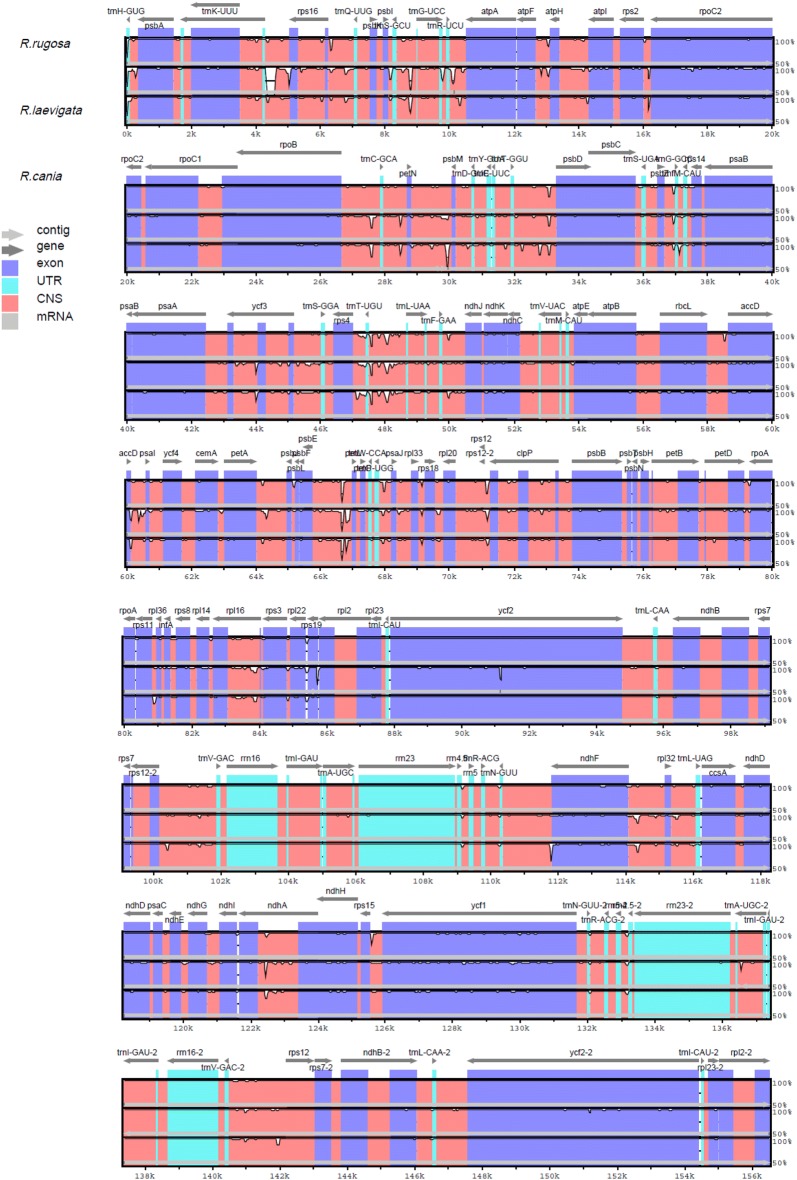
Comparative analyses of genomic differences in chloroplasts of *R. laevigata, R. rugosa* and *R. canina*. Gray arrows and thick black lines above the alignment indicate gene orientation. Purple bars represent exons, blue bars denote untranslated regions (UTRs), pink bars represent non-coding sequences (CNS) and gray bars denote mRNA. The y-axis represents the percentage identity

To ascertain differences in the genomic sequences of chloroplasts of *R. laevigata, R. rugosa* and *R. canina*, we used the sequence in *R. chinensis* as a reference (Fig. 4). Variability in the IR region of the cp genomes was considerably lower than that of LSC and SSC regions. In addition, most of the protein-coding genes of chloroplasts were highly conserved, except for the large variation in protein-coding genes of some genes (e.g., *rps19, petB*, and *ycf2*). Regions with a higher degree of variation among chloroplast genomic sequences were usually located in intergenic regions, such as the spacers for: trnH-GUU; *trnS*-*GCU* and *trnG*-*GCC*; *psbA*-*trnH*, *trnC*-*GCA* and *petN*; *trnT*-*GGU* and *psbD*; *petA* and *psbJ*; *ndhF* and *rpl32*; *psaC* and *ndhE*. Identification of such higher-resolution loci was necessary for use as barcodes for species identification.

### Comparison of IR regions in the cp genomes of *R. laevigata, R. rugosa, R. canina* and *R. chinensis*

**Fig. 5:**
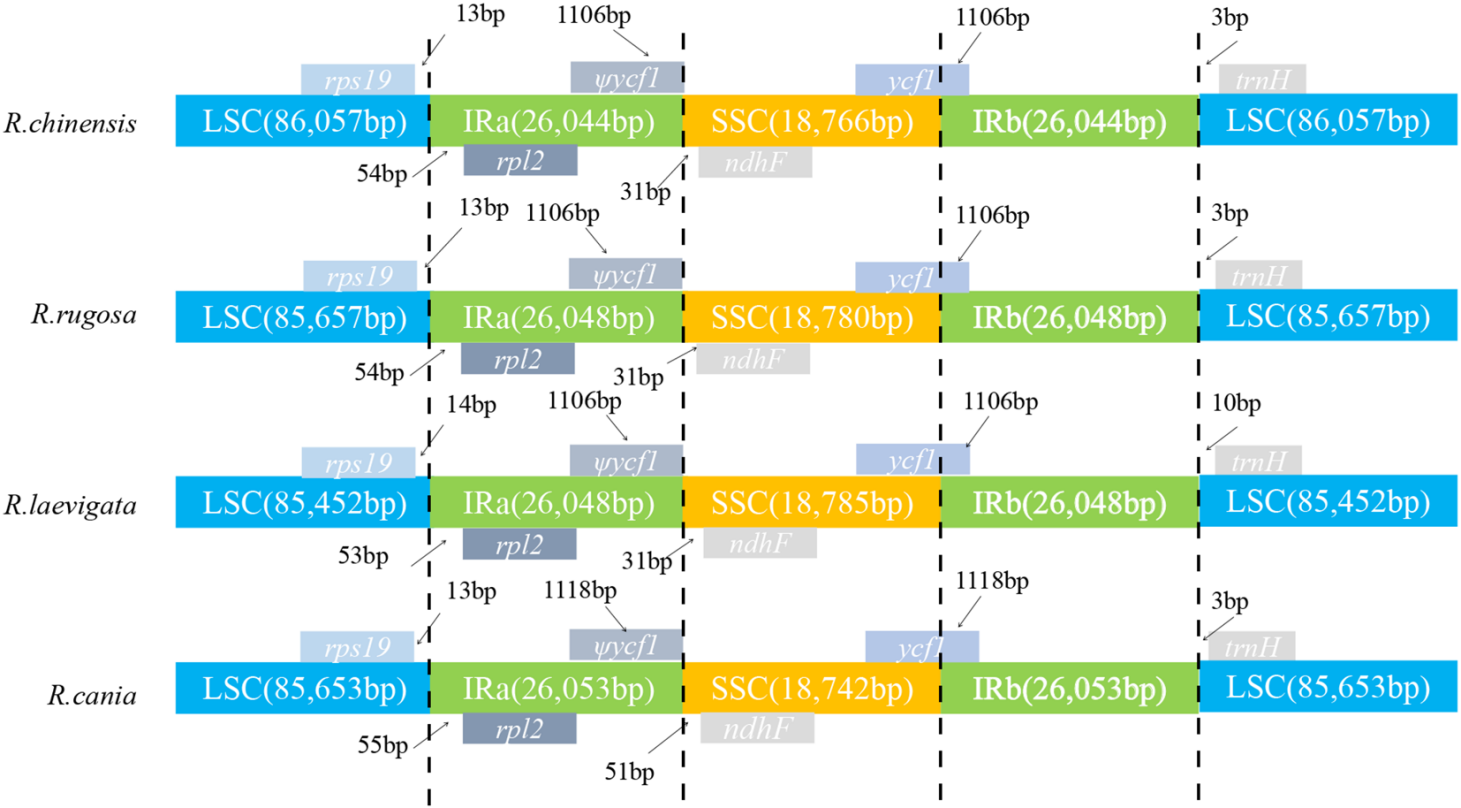
Comparison of genome boundaries in chloroplasts from *R. laevigata, R. rugosa*, *R. canina and R. chinensis*

Gene location was relatively conservative in *R. laevigata, R. rugosa*, *R. canina and R. chinensis*. In these four species, *rps19* was located in the LSC region, *rpl2* in the IRa region, and *ndhF* in the SSC region. However, the coding region of *ycf1* was at the border of SSC/IRb, and spanned the LSC region and IRb region, so the IRa/SSC boundary (5′ end was lost) region created a pseudogene. The region of mutations in the *ycf1* pseudogene in the IRa/SSC region was 1106–1118 bp (Fig. 5). The double-strand break repair theory is considered to be the main mechanism for expansion and contraction of the IR region. Large shrinkages of the IR region are relatively rare.

### Phylogenetic analyses

**Fig. 6:**
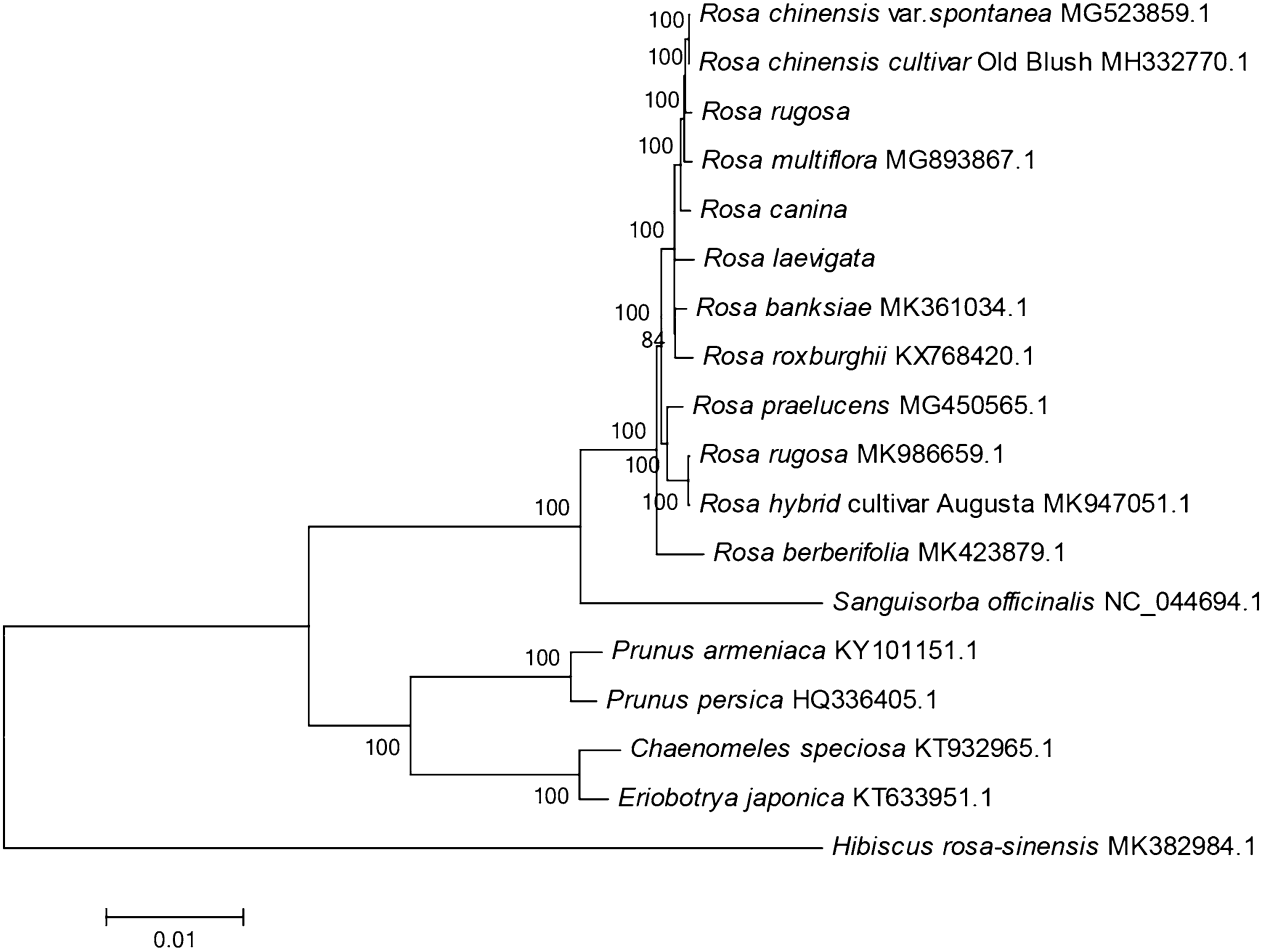
NJ tree based on the cp genomes of the Rosaceae family. *Hibiscus rosa*-*sinensis* was set as the outgroup

There have been many efforts to reconstruct the phylogenetic trees of plants of the genus *Rosa*. Several scholars have proposed that the extant classification system was artificial [12, 13], and that the interspecies relationships of *Rosa* are still ambiguous. The availability of the complete genomes of chloroplasts can provide further information for reconstruction of robust phylogeny for *Rosa*. A NJ tree was constructed for the cp genomes of 18 species of the Rosaceae family (Fig. 6). Species from the *Rosa* genus were monophyletic clade. Furthermore, *R. laevigata, R. rugosa*, *R. canina* and *R. chinensis* could be effectively divided into different sub-clades, and differentiated from each other efficiently. In which, *R. Chinensis* have a closer relationship with R. *rugosa*.

## Discussion

We identified the cp genomes of *R. laevigata, R. rugosa* and *R. canina* in this study, which are used in TCM formulations. The cp genomes of *R. laevigata, R. rugosa* and *R. canina* showed high similarities in terms of genome size, gene classes, gene sequences, codon usage, and distribution of repeat sequences. This is partly because of the extremely low levels of sequence divergence observed across the *Rosa* genus [54, 55]. Some intergenic regions were identified with high degree of variation, which will be used as barcodes for species identification. We also investigated introns in all anticipated genes of three *Rosa* species. Intron and/or gene losses have been reported for cp genomes [56–58]. Introns have important roles in regulation of gene expression [59], and they can control gene expression temporally and in a tissue-specific manner [60, 61]. Scholars have reported on the regulation mechanisms of introns for gene expression in plants and animals [62–64]. However, the connotations between intron loss and gene expression using the transcriptome for genus *Rosa* have not been published. More experimental work on the roles of introns shall be needed for future work. Comparative analysis of gene location in *R. laevigata, R. rugosa*, *R. canina and R. chinensis* revealed a pseudogene of *ycf1,* which may provide a basis for studying variations in the cp genomes of higher plants or algae.

Phylogenetic analyses revealed that Rosa genus belonged to monophyletic clade (Fig. 6), while their intra-family relationships were almost in agreement with those from a study by Zhang and Marie et al. [13, 65]. However, the exact phylogenetic location of some base taxons needs further verification, such as that the phylogenetic relationship of *R. rugosa* and *R. chinensis* in here contradicts what was previously reported, two *R. rugosa* species were clustered into two different clades. The possible reason is: complicates phylogeny reconstruction in roses was complicated by interspecific hybridization, some studies have suggested that there were frequent interspecific hybridization in the *Rosa* genus [11, 66–69]. Indeed, several contradictions between plastid and nuclear gene phylogenies of *Rosa* genus were discovered in previous study [55]. In addition, publications of numerous names given to morphological variants and hybrids, result in Rosa taxonomy further complication [70]. Further identification of plant material or sequencing of those hybrids could explain why conspecific samples sometimes fall into distinct clades [12].

## Conclusions

The whole cp genomes of *R. laevigata, R. rugosa* and *R. canina* was sequencing and analysis in this study. The status of the major taxa within the genus *Rosa* was consistent with our results for sequencing of cp genomes. *R. laevigata, R. rugosa*, *R. canina* and *R. chinensis* could be differentiated from other *Rosa* species efficiently. Our data reveal that cp genomes can be used for the identification and classification of *Rosa* species. Our results can aid studies on molecular identification, genetic transformation, and lay a theoretical foundation for the discovery of disease-resistance genes and cultivation of *Rosa* species. Our observations complement the database of herbgenomics [71].

## Data Availability

The datasets generated during the current study are available in the National Center for Biotechnology Information database (NCBI). https://www.ncbi.nlm.nih.gov/[MN661138, MN661139, MN661140].

## Abbreviations

LSC: Large single-copy
SSC: Small single-copy region
IR: Inverse repeat
cp: Chloroplast
NCBI: National Center for Biotechnology Information database
TCM: Traditional Chinese medicine

## References

[1] Anonymous. Pharmacopoeia of People’s Republic of China. Part 2. Chinese Medicine. Edited by Committee NP. Beijing: Science Press., 2015;75:200, 221.

[2] Wissemann V, Ritz CM (2005). The genus *Rosa* (Rosoideae, Rosaceae) revisited: molecular analysis of nrITS-1 and atpB-rbcL intergenic spacer (IGS) versus conventional taxonomy. Bot J Linn Soc.

[3] Christenhusz MJM, Fay MF, Chase MW (2018). Plants of the world: an illustrated encyclopedia of vascular plants. Madroño..

[4] Uzunçakmak T, Ayaz Alkaya S (2017). Effect of aromatherapy on coping with premenstrual syndrome: a randomized controlled trial. Complement Ther Med..

[5] Baiyisaiti A, Wang Y, Zhang X (2019). *Rosa rugosa* flavonoids exhibited PPARα agonist-like effects on genetic severe hypertriglyceridemia of mice. J Ethnopharmacol.

[6] Liu Y, Zhi D, Wang X, Fei D (2018). Rose (R Setate x R. Rugosa) decoction exerts antitumor effects in *C. elegans* by down regulating Ras/MAPK pathway and resisting oxidative stress. Int J Mol Med.

[7] Rehder A (1949). Bibliography of cultivated trees and shrubs hardy in the cooler temperate regions of the Northern Hemisphere.

[8] Zhu ZM, Gao XF, Fougère Danezan M (2015). Phylogeny of Rosa sections Chinenses and Synstylae (Rosaceae) based on chloroplast and nuclear markers. Mol Phylogenet Evol.

[9] Matthews JR (1920). Hybridism and classification in the genus rosa. New Phytol.

[10] Rowley G (1959). Some naming problems in rosa. Bulletin du Jardin botanique de l’Etat Bruxelles..

[11] Ritz CM, Schmuths H, Wissemann V (2005). Evolution by reticulation: European Dogroses originated by multiple hybridization across the genus *Rosa*. J Hered.

[12] Joly S, Starr JR, Bruneau A (2007). Phylogenetic relationships in the genus *Rosa*: new evidence from chloroplast DNA sequences and an appraisal of current knowledge. Syst Bot.

[13] Fougère-Danezan M, Simon J, Anne B (2015). Phylogeny and biogeography of wild roses with specific attention to polyploids. Ann Bot.

[14] Brunkard JO, Runkel AM, Zambryski PC (2015). Chloroplasts extend stromules independently and in response to internal redox signals. Proc Natl Acad Sci.

[15] Hu Y, Zhang Q, Rao G (2008). Occurrence of plastids in the sperm cells of *Caprifoliaceae*: biparental plastid inheritance in angiosperms is unilaterally derived from maternal inheritance. Plant Cell Physio..

[16] Daniell H, Kumar S, Dufourmantel N (2005). Breakthrough in chloroplast genetic engineering of agronomically important crops. Trends Biotechnol.

[17] Sigmon BA, Adams RP, Mower JP (2017). Complete chloroplast genome sequencing of vetiver grass (*Chrysopogon zizanioides*) identifies markers that distinguish the non-fertile ‘Sunshine’ cultivar from other accessions. Ind Crops Prod.

[18] Kim KJ, Lee HL (2004). Complete chloroplast genome sequence from Korean Ginseng (*Panax schiseng* Nees) and comparative analysis of sequence evolution among 17 vascular plants. DNA Res.

[19] Li P, Zhang S, Li F (2017). A phylogenetic analysis of chloroplast genomes elucidates the relationships of the six economically important brassica species comprising the triangle of U. Front Plant Sci..

[20] Shen X, Wu M, Liao B (2017). Complete chloroplast genome sequence and phylogenetic analysis of the medicinal plant *Artemisia annua*. Molecules.

[21] Li R, Ma PF, Wen J, Yi TS (2013). Complete sequencing of five Araliaceae chloroplast genomes and the phylogenetic implications. PLoS ONE.

[22] Yang JB, Li DZ, Li HT (2014). Highly effective sequencing whole chloroplast genomes of angiosperms by nine novel universal primer pairs. Mol Ecol Resour..

[23] Kane N, Sveinsson S, Dempewolf H (2012). Ultra-barcoding in cacao (Theobroma spp; Malvaceae) using whole chloroplast genomes and nuclear ribosomal DNA. Am J Bot.

[24] Dodsworth Steven (2015). Genome skimming for next-generation biodiversity analysis. Trends Plant Sci.

[25] Wang A, Wu H, Zhu X (2018). Species identification of *Conyza bonariensis* assisted by chloroplast genome sequencing. Front Genet..

[26] Luo H, Shi J, Arndt W (2008). Gene Order Phylogeny of the genus *Prochlorococcus*. PLoS ONE.

[27] Luo H, Sun Z, Arndt W (2009). Gene order phylogeny and the evolution of methanogens. PLoS ONE.

[28] Jeon JH, Kim SC (2019). Comparative analysis of the complete chloroplast genome sequences of three closely related east-asian wild roses (rosa sect. synstylae; rosaceae). Genes..

[29] Jiang HY, Zhang YH, Yan HJ (2018). The complete chloroplast genome of a key ancestor of modern roses, rosa chinensis var. spontanea, and a comparison with congeneric species. Molecules.

[30] Guo Q, Guo LL, Zhang L (2017). Construction of a genetic linkage map in tree peony (Paeonia Sect. Moutan) using simple sequence repeat (SSR) markers. Sci Hortic.

[31] Jiang H, Lei R, Ding SW (2014). Skewer: a fast and accurate adapter trimmer for next-generation sequencing paired-end reads. BMC Bioinform..

[32] Deng P, Wang L, Cui L (2015). Global identification of microRNAs and their targets in Barley under salinity stress. PLoS ONE.

[33] Gogniashvili M, Naskidashvili P, Bedoshvili D (2015). Complete chloroplast DNA sequences of Zanduri wheat (Triticum s). Genet Resour Crop Evol.

[34] Acemel RD, Tena JJ, Irastorza-Azcarate I (2016). A single three-dimensional chromatin compartment in amphioxus indicates a stepwise evolution of vertebrate hox bimodal regulation. Nat Genet.

[35] Boetzer M, Henkel CV, Jansen HJ (2010). Scaffolding pre-assembled contigs using SSPACE. Bioinformatics.

[36] Liu C, Shi L, Zhu Y (2012). CpGAVAS, an integrated web server for the annotation, visualization, analysis, and GenBank submission of completely sequenced chloroplast genome sequences. BMC Genomics..

[37] Wyman SK, Jansen RK, Boore JL (2004). Automatic annotation of organellar genomes with DOGMA. Bioinformatics.

[38] Lowe TM, Eddy SR (1997). tRNAscan-SE: a program for improved detection of transfer RNA genes in genomic sequence. Nucleic Acids Res.

[39] Lohse M, Drechsel O, Bock R (2007). OrganellarGenomeDRAW (OGDRAW): a tool for the easy generation of high-quality custom graphical maps of plastid and mitochondrial genomes. Curr Genet.

[40] Tamura K, Peterson D, Peterson N (2011). MEGA5: molecular evolutionary genetics analysis using maximum likelihood, evolutionary distance, and maximum parsimony methods. Mol Biol Evol.

[41] Frazer KA, Pachter L, Poliakov A (2004). VISTA: computational tools for comparative genomics. Nucleic Acids Res.

[42] Benson G (1999). Tandem repeats finder: a program to analyze DNA sequences. Nucleic Acids Res.

[43] Kurtz S, Choudhuri JV, Ohlebusch E (2001). REPuter: the manifold applications of repeat analysis on a genomic scale. Nucleic Acids Res.

[44] Beier S, Thiel T, Münch T (2017). MISA-web: a web server for microsatellite prediction. Bioinformatics.

[45] Choi KS, Park S (2015). The complete chloroplast genome sequence of *Aster spathulifolius* (*Asteraceae*); genomic features and relationship with *Asteraceae*. Gene.

[46] Guo S, Guo L, Zhao W (2018). Complete chloroplast genome sequence and phylogenetic analysis of *Aster tataricus*. Molecules.

[47] Doorduin L, Gravendeel B, Lammers Y (2011). The complete chloroplast genome of 17 individuals of pest species *Jacobaea vulgaris*: SNPs, microsatellites and barcoding markers for population and phylogenetic studies. DNA Res.

[48] Lee SB, Kaittanis C, Jansen RK (2006). The complete chloroplast genome sequence of *Gossypium hirsutum*: organization and phylogenetic relationships to other angiosperms. BMC Genomics..

[49] Saski C, Lee S, Daniell H (2005). Complete chloroplast genome sequence of Glycine max and comparative analyses with other legume genomes. Plt Mol Biol..

[50] Asaf S, Khan AL, Khan MA (2017). chloroplast genomes of *Arabidopsis halleri* ssp. gemmifera and *Arabidopsis lyrata* ssp. petraea: structures and comparative analysis. Sci Rep..

[51] Dong W, Xu C, Cheng T (2013). Sequencing angiosperm plastid genomes made easy: a complete set of universal primers and a case study on the phylogeny of Saxifragales. Genome Biol Evol..

[52] Yang Y, Zhou T, Duan D (2016). Comparative analysis of the complete chloroplast genomes of five Quercus species. Front Plant Sci..

[53] Suo Z, Li W, Jin X, Zhang H (2016). A new nuclear DNA marker revealing both microsatellite variations and single nucleotide polymorphic loci: a case study on classification of cultivars in *Lagerstroemia indica* L. J Microb Biochem Technol..

[54] Matsumoto S, Kouchi M, Yabuki J (1998). Phylogenetic analyses of the genus Rosa using the matK sequence: molecu-lar evidence for the narrow genetic background of modern roses. Scientia Hortic.

[55] Wissemann V, Ritz CM (2005). The genus Rosa (Rosoideae, Rosaceae) revisited: molecular analysis of nr ITS-1 and atpB-rbcL intergenic spacer(IGS) versus conventional taxonomy. Botanical J Linn Soc..

[56] Downie SR, Llanas E, Katz-Downie DS (1996). Multiple independent losses of the rpoC1 intron in angiosperm chloroplast DNAs. Syst Bot.

[57] Graveley BR (2001). Alternative splicing: increasing diversity in the proteomic world. Trends Genet.

[58] Ueda M, Fujimoto M, Arimura SI (2007). Loss of the rpl32 gene from the chloroplast genome and subsequent acquisition of a preexisting transit peptide within the nuclear gene in Populus. Gene.

[59] Xu J, Chu Y, Liao B (2017). *Panax ginseng* genome examination for ginsenoside biosynthesis. Gigascience..

[60] Le Hir H, Nott A, Moore MJ (2003). How introns influence and enhance eukaryotic gene expression. Trends Biochem Sci.

[61] Niu DK, Yang YF (2011). Why eukaryotic cells use introns to enhance gene expression: splicing reduces transcription-associated mutagenesis by inhibiting topoisomerase I cutting activity. Biol Direct..

[62] Callis J, Fromm M, Walbot V (1987). Introns increase gene expression in cultured maize cells. Genes Dev.

[63] Emami S, Arumainayagam D, Korf I (2013). The effects of a stimulating intron on the expression of heterologous genes in *Arabidopsis thaliana*. Plant Biotechnol J.

[64] Ted C, Manley H, Cornelia G (1991). A generic intron increases gene expression in transgenic mice. Mol Cell Biol.

[65] Zhang SD, Jin JJ, Chen SY (2017). Diversification of Rosaceae since the Late Cretaceous based on plastid phylogenomics. New Phytol.

[66] Joly S, Starr JR, Lewis WH (2006). Polyploid and hybrid evolutionin roses east of the Rocky Mountains. Am J Bot.

[67] Mercure M, Bruneau A (2008). Hybridization between the escaped Rosa rugosa(Rosaceae) and native *R. blanda* in Eastern North America. Am J Bot.

[68] Ritz CM, Koehnen I, Groth M (2011). To be or not to be the odd one out-allele-specific transcription in pentaploid dogroses (Rosa L. sect. Caninae (DC) Ser). BMC Plant Biol.

[69] Qiu XQ, Zhang H, Wang QG (2012). Phylogenetic relationships of wild roses in China based on nrDNA and matK data. Sci Hortic.

[70] Wissemann V, Roberts AV, Debener T, Gudin S (2003). Conventional taxonomy (wild roses). Encyclopedia of rose science.

[71] Hu H, Shen X, Liao B (2019). Herbgenomics: a stepping stone for research into herbal medicine. Sci China Life Sci..

